# Large-Scale SNP Discovery through RNA Sequencing and SNP Genotyping by Targeted Enrichment Sequencing in Cassava (*Manihot esculenta* Crantz)

**DOI:** 10.1371/journal.pone.0116028

**Published:** 2014-12-31

**Authors:** Wirulda Pootakham, Jeremy R. Shearman, Panthita Ruang-areerate, Chutima Sonthirod, Duangjai Sangsrakru, Nukoon Jomchai, Thippawan Yoocha, Kanokporn Triwitayakorn, Somvong Tragoonrung, Sithichoke Tangphatsornruang

**Affiliations:** 1 National Center for Genetic Engineering and Biotechnology (BIOTEC), National Science and Technology Development Agency (NSTDA), 113 Thailand Science Park, Pathum Thani, Thailand; 2 Institute of Molecular Biosciences, Mahidol University, Nakhon Pathom, Thailand; Shanghai Institutes for Biological Sciences, China

## Abstract

Cassava (*Manihot esculenta* Crantz) is one of the most important crop species being the main source of dietary energy in several countries. Marker-assisted selection has become an essential tool in plant breeding. Single nucleotide polymorphism (SNP) discovery via transcriptome sequencing is an attractive strategy for genome complexity reduction in organisms with large genomes. We sequenced the transcriptome of 16 cassava accessions using the Illumina HiSeq platform and identified 675,559 EST-derived SNP markers. A subset of those markers was subsequently genotyped by capture-based targeted enrichment sequencing in 100 F_1_ progeny segregating for starch viscosity phenotypes. A total of 2,110 non-redundant SNP markers were used to construct a genetic map. This map encompasses 1,785 cM and consists of 19 linkage groups. A major quantitative trait locus (QTL) controlling starch pasting properties was identified and shown to coincide with the QTL previously reported for this trait. With a high-density SNP-based linkage map presented here, we also uncovered a novel QTL associated with starch pasting time on LG 10.

## Introduction

Cassava (*Manihot esculenta* Crantz) is one of the most important crop species for energy supply with a total production of 262 million tonnes from over 20 million hectares of cultivated area worldwide [1]. Its starchy roots provide food for 800 million people globally and contribute significantly to food security. Cassava is often cultivated in marginal, low fertility land with uncertain rainfall under low intensity management. Its remarkable tolerance to abiotic stresses and adverse environments along with minimal requirement for fertilizers make cassava an attractive crop for resource-limited smallholder farmers.

Although cassava is traditionally grown by subsistence farmers, large-scale commercial planting has been established in response to agro-industrial processors in demand for starch. High starch content in cassava (20–40%) makes it an excellent energy source both for human consumption and biofuel applications [2]. Cassava starch can be extracted to high purity with less protein and other associated contaminants compared to starch from other tuber and cereal sources [3]. Starch viscosity is one of the important characteristics that determines the suitability of starch in each application [4], [5] and it is of interest to identify the quantitative trait loci (QTL) and candidate genes associated with this trait.

Cassava has a diploid chromosome number of *2n* = 36 and belongs to the family Euphorbiaceae. It is monoecious and predominantly outcrossing, rendering the species highly heterozygous. Vegetative propagation by mature woody stem cutting is commonly practiced, resulting in the lack of genetic diversity among the offspring, which often hinders crop development. Partly because it is grown in developing countries, far less research has been devoted to cassava compared to cereals [6]. The potential for genetic improvement in cassava is still substantial [7]. Unfortunately, sporadic flowering pattern, low seed set and a long breeding cycle present significant barriers to the process of improving yield and disease resistance by conventional breeding. Marker-assisted selection has played a pivotal role in accelerating agricultural breeding programs [8]. The availability of molecular markers is essential for the utilization of genomic tools in cassava breeding. A number of molecular markers have been developed (restriction fragment length polymorphisms, amplified fragment length polymorphisms, and simple sequence repeats (SSRs)) and used to construct genetic linkage maps [9]–[13]. In the past decade, researchers have primarily relied upon SSR markers; however, their sparse distribution across the genome and the labor-intensive genotyping approach render them less than ideal for fine-mapping and large-scale genotyping assays.

Single nucleotide polymorphisms (SNPs) have recently become markers of choice for high-density genetic mapping owing to their sheer abundance in the genome [14]. SNPs are known to occur at frequencies of one per ∼100–500 bp in plant genomes, depending on the species, e.g. 1 SNP/121 bp in cassava [15], 1 SNP/204 bp in maize [16], and 1 SNP/500 bp in Arabidopsis [17]. Rapid advancement in sequencing capability together with the reduction in sequencing cost allow for effective genome-wide discovery of SNPs. For organisms with large genomes such as cassava (770 Mb; [18]), transcriptome sequencing (RNA-seq) provides an efficient means to restrict the sequencing to the expressed portion of the genome while still identifying a large amount of genetic variation [19]. Substantial improvement on genomic resources for cassava, largely achieved through the sequencing of the cassava genome [20], [21], greatly facilitates the characterization of variability within a crop by high throughput re-sequencing. RNA-seq has successfully been applied to large-scale SNP discovery and EST-derived SNP development in various plant species [22]–[26].

Despite the continual reduction in sequencing costs driven by next-generation sequencing, whole-genome sequencing for multiple individuals remains costly for species with large genomes. One approach that has successfully been applied to obtain reduced representations of the genome is targeted enrichment sequencing [27]. Using this strategy, probes complimentary to the target regions of the genome are designed and hybridized to genomic DNA for sequence capture and subsequent sequencing. This technique, together with the RNA-seq based SNP discovery, allows the predefined regions encompassing EST-SNPs to be selectively captured and sequenced in multiple individuals [28]. By focusing on pre-selected regions in the genome, targeted enrichment sequencing also enhances the coverage at the desirable marker loci, increasing the power to identify variants. Other available reduced representation sequencing strategies, such as restriction site-associated DNA sequencing (RAD-seq) and genotyping-by-sequencing (GBS), involve the use of restriction enzymes to secure similar genomic fractions from different individuals to screen for polymorphic markers [29]. The advantages of these restriction enzyme-based techniques are their ability to simultaneously discover and genotype genomic derived SNPs and their low cost of operation. However, unlike the targeted enrichment sequencing, these approaches do not allow the enrichment of specific target sequences in the genome. We employed sequence capture as our complexity reduction method since we would like to genotype SNP markers located specifically in the coding regions, which represent only 5.24% of the genome sequence [21].

The goals of this study were to perform a transcriptome-wide identification of EST-derived SNP markers from 16 cassava accessions and to utilize them to genotype a cassava mapping population using targeted enrichment sequencing. We demonstrated the feasibility of generating a high-density genetic map and rapidly locating major QTLs affecting starch viscosity using a relatively small mapping population. Our work represents the first effort to perform capture-based targeted enrichment sequencing in cassava and illustrates the attractiveness of this approach for genotyping SNPs in predetermined genomic regions.

## Material and Methods

### Plant material and field experiments

For RNA isolation, young leaf tissues (shoot apical meristems) were collected from cassava cultivars, wild Manihot relative (*Manihot glaziovii*) and other Manihot accessions maintained at the Rayong Cassava Center's experimental field. The permits to enter the field locations at Rayong Field Crop Research Center (12°44′06.8″N 101°08′09.4″E) and Lop Buri Crop and Production Resources Technical Service Center (15°22′02.0″N 100°55′01.6″E) were issued by Field Crops Research Instititute, Department of Agriculture, Thailand. These locations are not privately owned, and the field studies did not involve protected or endangered species. The samples used for transcriptome sequencing are as follows: (1) Rayong 60 (2) Rayong 3 (3) CM 323–375 (4) V13 (5) SV7-19-3K (6) CMH 22-77-1 (7) CMR 34-44-40 (8) CMK 23-27-30 (9) V4C (10) MCOL638 (11) MGUA22 (12) MCOL912B (13) MCUB23 (14) Hanatee (15) Huay Bong 60 (the first 15 samples are *M. esculenta*) and (16) *M.glaziovii.* Leaf samples were frozen in liquid nitrogen immediately after harvesting and kept at −80°C until RNA extraction.

A total of 100 F_1_ progeny from a cross between Thailand's two commercial lines Huay Bong 60 (female) and Hanatee (male) was used as a mapping population. Planting methods and phenotypic evaluation of this population were previously reported in Thanyasiriwat et al. [30]. Briefly, the F_1_ seedlings were planted at Rayong Field Crop Research Center in 2007, with a planting distance of 1 m between plants and 1.5 m between rows. (No replication was carried out for the 2007 field trial.) The phenotypes were evaluated 12 months after planting. In 2008, 100 F_1_ progeny were planted in two locations: Rayong Field Crop Research Center and Lop Buri Crop and Production Resources Technical Service Center. Each genotype was replicated twice in a simple lattice design, with 1 m between plants and 1 m between rows. Phenotypic evaluations were performed at both locations 12 months after planting (in 2009). Leaf tissues were collected from the two parents and the progeny, and kept frozen until DNA extraction.

### RNA extraction and transcriptome sequencing

Total RNA was extracted from 16 cassava accessions using the PureLink Plant RNA Reagent (Life Technologies, Grand Island, NY, USA) and treated with DNase I to remove residual DNA contaminant. RNA samples were purified using the RNeasy Mini Kit (Qiagen, Hilden, Germany) and subjected to quantity and quality assessment by the BioAnalyzer 2100 (Agilent Technologies, Santa Clara, CA, USA). Prior to cDNA library construction, we enriched the RNA samples for transcripts using the Absolutely mRNA Purification Kit (Agilent Technologies, Santa Clara, CA, USA). The cDNA libraries were constructed and Illumina paired-end adapters and barcode sequences were ligated onto the cDNA fragments. The pooled libraries were sequenced at Macrogen (Seoul, Korea) using the Illumina HiSeq2000 platform (Illumina, San Diego, CA, USA).

### RNA-Seq data analysis and SNP calling

Sample separation and adapter/barcode trimming were performed using the standard Illumina software and the quality of the trimmed reads was checked with FastQC. The RNA-Seq reads were aligned to the cassava reference genome (Cassava v5.0 assembly; [21]) with the general feature format (GFF) file [20] using TopHat v2.0.9 calling Bowtie2 v2.1.0 [31], [32]. TopHat and Bowtie2 were run using default settings, under which ambiguous alignments were randomly assigned to one of their possible positions in the genome. The Genome Analysis Toolkit Unified Genotyper (GATK; version 2.8-1-g932cd3a) was used to call SNPs in the 16 accessions, resulting in a multi-sample variant call format (VCF) file [33]. Default parameters were used for GATK, which assumed a heterozygosity rate of one every 1000 bp. Variant calls with a quality less than 20 were subsequently removed with the VCF filter.

### Analyses of synonymous and non-synonymous changes in RNA-seq

We determined the types of mutations from the RNA-seq data brought about by single nucleotide variations in the coding regions using the program SNPEff v3.4e [34] with cassava reference genome sequence and GFF annotation input files. Only genes that had a mean read depth of at least 20 for all 16 accessions were included in the analyses.

### DNA extraction, probe design and targeted enrichment sequencing

The set of polymorphisms identified in the RNA-seq data was filtered to identify SNPs that were informative for a cross between Huay Bong 60 and Hanatee. A total of 27,469 biallelic SNPs from 10,105 regions were used to design probes for sequence capture in genomic DNA. Probes were designed and synthesized by Life Technologies for the Ion TargetSeq Custom Enrichment Kit (Life Technologies, Grand Island, NY, USA) to target a total of 2.49 Mb of genomic DNA.

The genotyping was performed with two parental strains (Huay Bong 60 and Hanatee) and 100 segregating progeny. Frozen leaf tissues were homogenized in liquid nitrogen, and DNA was extracted using the DNeasy Plant Mini Kit (Qiagen, Hilden, Germany). The integrity of the DNA was verified by the BioAnalyzer 2100 (Agilent Technologies, Santa Clara, CA, USA). For targeted enrichment sequencing, 500 ng of each DNA sample were fragmented enzymatically using the Ion Shear Plus Reagents (Life Technologies, Grand Island, NY, USA) and ligated to the adapters containing specific barcodes. Samples were multiplexed after the adapter-barcode ligation step in order to reduce the cost of subsequent hybridization and sequencing. Hybridization with the custom designed capture probes (see **Results and discussion** for details) was carried out according to the Ion TargetSeq Custom Enrichment Kits protocol to enrich sheared genomic fragments for regions of interest. The adapter-ligated, enriched fragments were used to construct a library, which was then sequenced on the Ion Torrent Proton System (Life Technologies, Grand Island, NY, USA) using the PI chip.

### SNP calling in a mapping population

Raw reads were de-multiplexed according to their barcodes and the adapter/barcode sequences were removed using the standard Ion Proton software. Clean reads were mapped to the cassava reference genome using Tmap and the variants were called using the GATK (version v1.4-749-g8b996e2) as described above (see **RNA-Seq data analysis and SNP calling**). SNPs that had an unexpected pattern of inheritance (e.g. offspring possessing alleles not present in either of the parents or offspring inheriting both alleles from a single parent) were filtered out. We then selected for SNP markers that were informative in this population, i.e. at least one of the parents had to be heterozygous. Finally, only SNP positions that were located within the target regions were used for genetic analysis.

### Construction of the linkage map

Polymorphic markers were classified into two categories according to their segregation patterns. The test-cross markers (heterozygous in one parent and homozygous in the other; AA×AB or AB×BB) segregated in a 1∶1 ratio while the inter-cross markers (heterozygous in both parents; AB×AB) segregated in a 1∶2∶1 ratio. Markers that deviated significantly from the expected Mendelian segregation ratios (χ^2^ test *p*-value <0.01) were removed from further analysis. Filtered SNPs (for the criteria mentioned above) were used to construct a linkage map using JoinMap v3.0 [35] with a LOD score threshold of 7. Markers with more than 10% missing data were excluded from map construction. The map was plotted using the R package ggbio [36].

### QTL analysis

QTL analysis was performed with the software MapQTL 4.0 [37], using an interval mapping function. The genome-wide and chromosome-wide LOD thresholds were determined by 1000 permutations using the α = 0.05 threshold [38]. The LOD plots for the linkage groups on which significant QTLs were identified were graphically presented using the R package ggbio [36]. The QTL was determined as significant if its LOD score was higher than the genome-wide threshold and was considered suggestive if the LOD score was between the genome-wide and the chromosome-wide thresholds.

## Results and Discussion

### Transcriptome sequencing and SNP discovery

To develop a large number of genic SNP markers across the genome, we sequenced the transcriptome from 16 cassava accessions maintained at the Cassava Research Center, Thailand. A total of 407,327,102 cleaned reads covering 41.14 Gb were obtained from RNA-seq of the 16 accessions after the removing low-quality reads and adapter/barcode trimming. The number of reads was distributed evenly among the 16 samples and, on average, 95.70% of all the bases had quality scores greater than 20 (Table 1).

**Table 1:** Statistics for RNA-seq data from 16 cassava accessions.

Species	Accessions	Total bases	Read counts	% Mapped reads	GC content (%)	% of total bases with Q20
*M.esculenta*	Rayong 60	2,632,033,336	26,059,736	82.5	44.50	95.69
	Rayong 3	3,017,714,562	29,878,362	83.0	44.85	95.96
	CM323–375	2,815,016,854	27,871,454	83.2	44.47	95.89
	V13	1,844,070,322	18,258,122	82.9	44.49	95.95
	SV7-19-3K	2,565,795,718	25,403,918	82.6	44.39	95.62
	CMH22-771	2,483,078,334	24,584,934	81.8	44.66	95.40
	CMR34-44-40	2,439,871,140	24,157,140	84.2	44.16	96.13
	CMK23-2730	2,691,600,914	26,649,514	82.6	44.89	95.88
	V4C	2,600,062,594	25,743,194	80.9	44.62	95.62
	MCOL638	2,530,090,400	25,050,400	81.9	44.76	95.65
	MGUA22	2,621,654,980	25,956,980	83.6	44.03	95.83
	MCOL912B	2,413,738,602	23,898,402	83.9	44.55	95.86
	MCUB23	2,803,467,100	27,757,100	84.1	44.56	95.61
	Hanatee	2,534,735,188	25,096,388	74.6	45.79	95.62
	Huay Bong 60	2,676,322,644	26,498,244	63.8	46.85	95.14
*M.glaziovii*		2,470,784,614	24,463,214	69.3	44.78	95.40

Quality scores measure the probability that the base is called incorrectly. Quality scores of 20 (Q20) represent a corresponding call accuracy of 99%.

To identify polymorphisms among different accessions, each RNA-seq read was first mapped against the Cassava v5.0 genome assembly using TopHat2. On average, 80% of the reads from each accession were able to align to the reference genome (Table 1). We identified a total of 698,347 genetic variations in 16 cassava accessions. Of those, 675,559 were single nucleotide substitutions and 22,788 were insertion/deletion (indel) polymorphisms. The proportion of indels discovered here (3.26%) is slightly lower than the number previously reported (6%) in Sakurai et al. [39], which utilized the public EST database to identify putative SNP markers. The error associated with calling indels from our Illumina read alignment might be lower than that of the EST alignment since we had significantly higher depth coverage across the transcriptome. We focused only on single nucleotide variations and excluded the indels because they were more likely derived from sequencing errors and/or misalignment. The entire transcriptome sequence (30,666 protein-coding loci) was covered by at least one read from all 16 samples, and 27,402 transcripts (89% of all transcripts) were covered by at least 50 raw reads.

The frequency of single nucleotide substitutions found in the cassava transcriptome was approximately 1 in 350 nucleotides, which was within the ranges previously reported for cassava (1 SNP/121 bp in [15], 1 SNP/509 bp in [40], 1 SNP/1,072 bp in [39]). The SNP frequency reported in Kawuki et al. [15] was much higher than the frequency observed here. This was anticipated as the number of accessions employed in Kawuki et al. [15] was higher than the number of genotypes investigated in this study. Additionally, the genetic base of our 16 accessions appeared to be narrower than that of the 74 genotypes examined in [15], leading to a lower frequency of SNP discovery here.

Of the 698,347 variations discovered, 377,995 (54%) were transitions (C/T or A/G) and 293,925 (42%) were transversions (A/C, A/T, C/G or G/T), with the A↔G transition being the most prevalent (27%) and the C↔G transversion being the least common (8%) variation (Table 2). The transition/transversion ratio was 1.29, which was comparable to the previously published numbers (1.24 in [39] and 1.27 in [26]). This ratio was slightly lower than those reported in rubber tree (1.67), oil palm (1.78), sunflower (1.72) and eggplant (1.65) [41]–[44]. The bias in transition/transversion ratios commonly observed in SNP discovery probably reflects the frequent incidence of spontaneous deamination of 5-methylcytosine to thymine in the genome [45]. The degeneracy of the genetic code and the selective pressure for gene conservation are likely accountable for the dominance of transitions over transversions. Synonymous substitutions are more often transitions than transversions, and there is a stronger selection against replacement substitutions, leading to higher occurrences of transitions [46].

**Table 2:** Summary of polymorphisms identified in 16 cassava accessions.

Total number of polymorphisms:		698,347	100%
**Indels:**		**22,788**	**3.26%**
**SNPs:**		**675,559**	**96.74%**
*Bi-allelic:*		671,920	96.22%
	Transition	377,995	54.13%
	A/G	189,591	27.15%
	C/T	188,404	26.98%
	Transversion	293,925	42.09%
	A/C	71,826	10.29%
	A/T	92,841	13.29%
	C/G	57,532	8.24%
	G/T	71,726	10.27%
*Tri-allelic:*		3,624	0.52%
*Quad-allelic:*		15	0.002%

### Analysis of synonymous and non-synonymous SNPs in cassava genes

We employed the SNPEff software to identify nucleotide substitutions that result in changes in amino acid (i.e. non-synonymous) and those that do not (i.e. synonymous) [34]. Among 409,847 biallelic SNPs identified within the exonic regions (the remaining were located in the introns or 5′/3′-untranslated regions), roughly half (53%) were synonymous (silent) mutations, and the majority of non-synonymous substitutions were non-conservative mutations (Table 3). The percentage of non-synonymous substitutions found in cassava (47%) was similar to those reported in tomato (46%) [47] and Arabidopsis (45%) [48]. Those SNPs were further classified based on the variant position within the codons. The majority of the genic SNPs were located in the third codon position, consistent with the hypothesis that deleterious mutations occurring at the first or second base, which likely result in non-synonymous substitutions, have been selected against and eventually eliminated during the course of evolution [47].

**Table 3:** Analysis of synonymous and non-synonymous changes in cassava SNPs.

Total number of exonic SNPs:	409,847	100%
**Position in the codon:**		
1^st^ base of the codon	97,763	23.85%
2^nd^ base of the codon	79,354	19.36%
3^rd^ base of the codon	232,730	56.78%
**Synonymous mutations:**	**217,594**	**53.09%**
**Non-synonymous mutations:**	**192,253**	**46.91%**
Missense:		
Conservative	66,072	16.12%
Non-conservative	124,747	30.43%
Nonsense:	1,133	0.28%
Read-through:	301	0.07%

Detailed information regarding the changes introduced by nucleotide substitutions and their distribution according to the nucleotide position within codons.

### Probe design for targeted enrichment sequencing

For large and complex genomes, it is still costly to sequence the entire genome with sufficient depth coverage to reliably identify SNPs. Targeted sequence enrichment is an attractive approach designed to isolate a specific genomic fraction for subsequent next-generation sequencing, improving depth coverage of the targeted regions. To demonstrate the feasibility of a rapid QTL mapping strategy that combines targeted enrichment with genotyping-by-sequencing, we selected a subset of SNP markers to genotype the population of 100 F_1_ progeny derived from a cross between two commercial lines, Huay Bong 60 and Hanatee. From a total of 675,559 biallelic SNPs identified in 16 cassava accessions, 449,895 positions were called in both Huay Bong 60 and Hanatee (i.e. no missing data) and 150,608 positions were polymorphic between the two cultivars. After excluding polymorphic markers for which Huay Bong 60 and Hanatee were both homozygous (the markers in AA×BB configuration would yield a uniform, non-segregating AB genotype in all F_1_ offspring), there were a total of 118,818 informative SNP loci. We subsequently filtered out SNP markers that exhibited the minor allele frequency (MAF) less than 0.1 for AA×AB configuration or less than 0.3 for AB×AB configuration. Finally, we selected 27,469 SNP sites in single copy genes for the proprietary probe design for the Ion TargetSeq Custom Enrichment Kit (Life Technologies, Grand Island, NY, USA). The distribution of the capture probes along the pseudo-chromosomes (Cassava v5.0 assembly [21]) is illustrated in S1 Fig.

### SNP genotyping with sequence capture

Genomic DNA from 102 individuals (two parental strains, Huay Bong 60 and Hanatee, and 100 F_1_ progeny) was enriched using the capture probes prior to being sequenced in multiplex on eight Ion Proton PI chips. We obtained an average of 5.1 million reads per sample and the mean read length of 136 bases (S1 Table). On average, 78% of the total bases sequenced had a quality score of 20 or higher. Alignment of adapter-trimmed reads to the cassava genome revealed a high degree of on-target enrichment efficiency. Approximately 80% of the reads mapped directly to the target regions covered by the capture probes, suggesting that the hybridization of the target probes binding to off-target fragments was infrequent. The distribution of the mapped reads across the targeted regions was highly uniform, with an average of 95.4% uniformity. We also assessed the efficiency of hybridization between target DNA and captured probes. The majority of the bait regions (97%) were covered by at least 50 uniquely mapped reads and only 0.36% of the target regions were not covered by any reads.

The sequence reads from 102 lines were mapped to the cassava reference genome using Tmap and the SNP genotypes in each individual were called. A total of 55,023 informative SNP markers could be called unambiguously with a mean read depth (across all samples and SNPs) of 145±74. Because a single targeted SNP was spanned by ∼150 bases of capture probes on either side, we acquired an additional number of non-targeted SNPs within the bait regions. Those non-targeted SNPs were omitted in the original SNP set due primarily to insufficient depth coverage at those loci (in the RNA-seq experiment). Out of the total 55,023 markers, 42,032 SNPs were located in the coding regions and 9,675 were in the introns. Additionally, 1,517 and 799 non-targeted SNPs were discovered in the untranslated and intergenic regions, respectively. Although the DNA sequences captured extended beyond both ends of the target probes (because the median length of sheared DNA fragments was greater than the length of the capture baits), we were not able to call any SNPs from these adjacent regions due to inadequate/inconsistent depth coverage between samples.

### Construction of a linkage map

**Figure 1:**
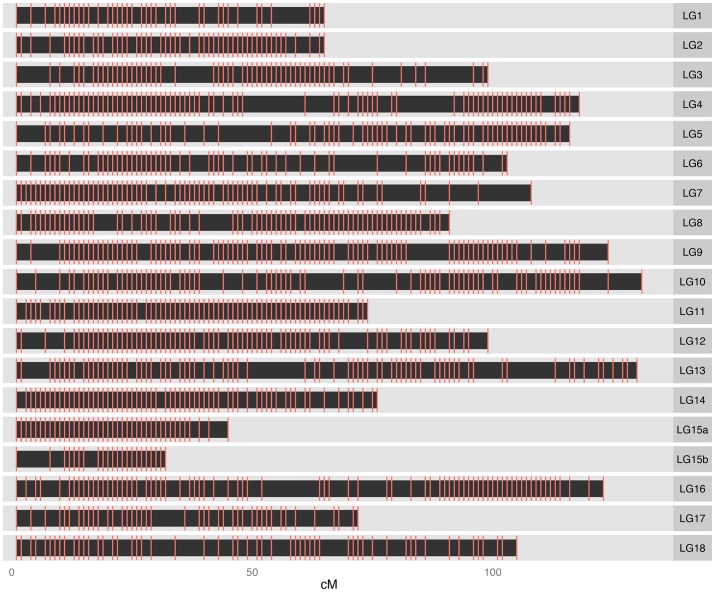
High-density genetic map of cassava. The SNP-based map included 2,110 non-redundant markers distributed over 19 linkage groups (LGs). The length of the each linkage group (in cM) was indicated at the bottom.

The Chi-square analyses revealed that 34,822 SNP markers followed Mendelian segregation ratios (χ^2^ *p*-value <0.01). Markers exhibiting perfect co-segregation were removed from the data prior to map construction. The genetic map of the F_1_ population comprised 2,110 non-redundant SNP markers distributed on 19 linkage groups (Fig. 1). The current map encompassed 1,785.62 cM, with the linkage groups ranging from 131.17 cM (LG 10) to 32.97 cM (LG 15b) in length (Table 4). The availability of the new assembly release, Cassava v5.0 [21], in which a large number of scaffolds were anchored into 18 chromosomes, allowed the comparisons between the genetic and physical maps. The linkage groups were assigned the corresponding chromosome numbers (Table 4). Each chromosome, with an exception of chromosome XV, has a single corresponding linkage group. Our inability to join two linkage groups (LG 15a and LG 15b) belonging to chromosome XV was probably due to a small mapping population size used to generate the genetic map. The total length of this SNP-based map (1,785 cM) is comparable to those of the previously published SSR-based maps (1,837 cM [49]; 1,420 cM [50]). Given the estimated genome size of 770 Mb of *M. esculenta* [18], the average recombination rate across all the linkage groups was 2.32 cM/Mb. The recombination rates in photosynthetic species vary from 0.75 cM/Mb in maize, ∼4 cM/Mb in rice and *Arabidopsis thaliana*, to 9.15 cM/Mb in *Chlamydomonas reinhardtii* [51].

**Table 4:** Distribution of SNP markers on the linkage groups.

Linkage group	Chromosome	No. of SNP markers	Length (cM)	Average distance between markers (cM)
LG 1	I	66	65.15	1.02
LG 2	II	84	65.75	0.79
LG 3	III	111	99.58	0.91
LG 4	IV	119	118.15	1.00
LG 5	V	95	116.73	1.24
LG 6	VI	91	103.81	1.15
LG 7	VII	165	108.15	0.66
LG 8	VIII	129	91.89	0.72
LG 9	IX	162	124.70	0.77
LG 10	X	114	131.17	1.16
LG 11	XI	150	74.72	0.50
LG 12	XII	147	99.19	0.68
LG 13	XIII	125	130.27	1.05
LG 14	XIV	140	76.04	0.55
LG 15a	XV	104	45.24	0.44
LG 15b	XV	38	32.97	0.89
LG 16	XVI	126	123.74	0.99
LG 17	XVII	69	72.76	1.09
LG 18	XVIII	75	105.61	1.45
Average	-	111.05	93.98	0.85
Total	-	2,110	1,785.62	-

The average number of SNP markers mapped to each linkage group was 111 (Table 4). The mean distance between markers was 0.85 cM across 19 linkage groups, with 72% of the intervals smaller than this average. Because the number of markers incorporated into this map was much higher than that used in Sraphet et al. [11], the average inter-marker distance observed here (0.85 cM) was significantly smaller than the previously reported distance (4.54 cM). With a myriad of SNP markers developed here, a genetic map with higher resolution can readily be achieved upon increasing the number of recombination events examined.

Regions of uneven and non-random marker distributions were observed on LG 4, LG 13 and LG 16 (Fig. 1). Since the SNP markers were identified from the RNA-seq experiment, those gaps may represent gene-poor, heterochromatic regions that are interspersed in the genome. These regions are often suppressed for meiotic crossovers and exhibit reduced recombination rates [52]. Non-uniform distribution of genes on chromosomes seems to be a common feature in higher eukaryotes [53], [54]. Genes are unevenly distributed on plant chromosomes. In *A. thaliana*, only 45% of the genome accounts for all 25,000 genes [55]. Gene-rich and gene-poor regions have also been observed in pea, date palm and tomato [56]. Large and poorly resolved gaps may also represent the centromeric regions, which often contain hundreds of kilobases of simple repeats and retroelements [57]. Another possible explanation for the occurrence of large intervals in the linkage groups is that the coverage of capture probes in those regions might be sparse.

To analyze the consistency of our map, we conducted a comparison between the marker order on our linkage map and their positions on the genome (using the Cassava v5.0 assembly as a reference [21]). A vast majority of the SNPs on the genetic map displayed conserved assignments with their corresponding physical locations (S2 Table). Occasionally, we observed a discrepancy in the marker order on the linkage map and their physical positions on the genome, particularly between closely mapped markers. This is because of the low number of the recombination breakpoints we examined in our mapping population. The accuracy of marker order on a genetic map generally improves with increasing population size.

### QTL analysis of starch viscosity traits and candidate genes identified

**Figure 2:**
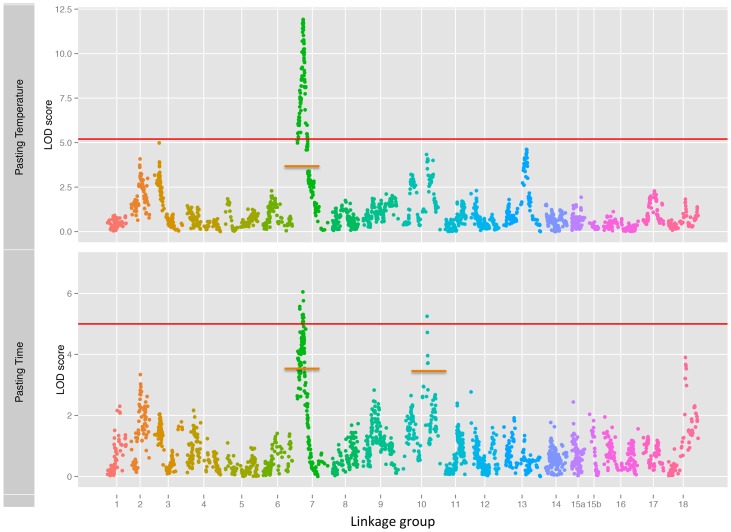
Manhattan plot of QTL LOD scores for starch viscosity traits. The Manhattan plots display the LOD scores of SNP markers along each linkage group (designated at the bottom). The red and orange lines indicated genome-wide and chromosome-wide significance thresholds, respectively.

Cassava starch is used in a variety of food products and non-food industrial applications. The starch pasting properties are the important characteristics that determine the suitability of starch in each application and have a great impact on the product quality [5]. In order to demonstrate the utility of capture-based enrichment sequencing for QTL mapping with a relatively small population, we applied the targeted enrichment sequencing technique to genotype 100 F_1_ progeny segregating for starch viscosity. The interval mapping detected a single, co-localized QTL for pasting temperature and pasting time across three environments on LG 7 (Fig. 2). The major QTL controlling starch pasting temperature had a LOD score of 12 and explained 44.7% of the total phenotypic variation observed while the QTL regulating starch pasting time had a LOD score of 6 and explained 24.3% of total phenotypic variations. Both of these QTLs coincide with the positions of the QTLs discovered in previous publication [30]. Additionally, we detected a novel QTL associated with starch pasting time on LG 10. This QTL accounted for 22.5% of the phenotypic variations across three environmental conditions, and it has not been identified in previous mapping attempts possibly due to insufficient marker coverage of this region on earlier SSR-based linkage maps. This greatly emphasizes the benefit of high-density linkage maps in facilitating a discovery of novel QTL.

Because transcript-derived SNPs are located within transcribed regions, they have a higher probability, compared to SNPs identified from genomic sequences, of being the underlying polymorphisms in the causative genes or QTLs. Several SNP markers with the LOD score ≥11.0 were associated with the QTL contributing a major effect on pasting temperature (S2 Table). Two closely linked genes involved in the synthesis of starch were in the vicinity of those markers: starch synthase 4 (cassava4.1_003800m) and sucrose synthase 6 (cassava4.1_001874m). Interestingly, the nucleotide variation found in each of these genes led to a non-synonymous change in the protein-coding region. The starch synthase 4 has a nucleotide substitution that alters Met-147 to Ile. According to the local alignment with the NCBI protein database, residue 147 of this polypeptide seems to be in the glycogen synthase domain. The Arabidopsis mutant carrying a T-DNA insertion in starch synthase 3 gene exhibited significant changes in its starch granule morphology [58]. It is possible that the M147I mutation could affect the structure of the starch granule, which in turn alters the starch pasting viscosity.

The sucrose synthase 6 has a nucleotide substitution that leads to a non-conservative alteration of the encoded protein, changing amino acid 573 from Lys to Ser. This residue appears to be in the C-terminal domain exhibiting glycosyltransferase activity [59]. From the crystal structure analysis of the Arabidopsis sucrose synthase 1, its Lys-585 residue played a role in substrate binding [59]. Given the degree of similarity between sucrose synthase in cassava and Arabidopsis, a mutation at Lys-573 in cassava's sucrose synthase 6 could affect the activity of the enzyme, leading to the observed pasting viscosity phenotype.

This study demonstrated that a pilot mapping project with a small number of progeny can be initiated to narrow down the regions containing (major) QTLs responsible for the traits of interest. We were able to identify the major QTL controlling starch pasting temperature and pasting time with only 100 F_1_ individuals genotyped. This preliminary approach can be followed by a more thorough fine-mapping to pinpoint the exact location of the QTLs. Targeted enrichment sequencing with the same set of capture probes can be applied to different cassava population for QTL mapping of other desirable traits or to the germplasm for phylogenetic studies. The apparent drawback of this method is that the minor QTLs may not be revealed as the power of detection is reduced with fewer number of recombination events sampled. Increasing the number of progeny genotyped will improve the chance of detecting minor QTLs.

For plant species possessing large genomes, it may not be cost-effective to sequence the entire genome from multiple individuals in a mapping population. Reduced representation methods are extremely useful, not only because of their cost-reducing aspects, but also because many research questions can be answered with a small set of markers and do not require every base of the genome to be sequenced [60]. There are several techniques employed to reduce genome complexity and to capture only a fraction of the genome to be sequenced. Restriction enzyme-based methods, such as RAD-seq and GBS, provide an unbiased approach to discover genome-wide sets of markers [61]. Even though those techniques maybe more cost-effective than the sequence capture-based methods, they do not target particular regions of the genome. If the regions of interest are known, they can be targeted directly with sequence capture methods [62]. In our case, targeted enrichment sequencing allows us to focus primarily on EST-derived SNPs, which can be powerful in detecting the causative mutations. Finally, the sequence capture approach is highly accurate when a high-quality reference sequence is available.

## Conclusions

Our study describes the large-scale, transcriptome-wide identification of over 698,000 single nucleotide variations from the transcriptome of 16 cassava genotypes. We discovered that approximately 3% of the variations are indels while the rest represent single nucleotide substitutions. From this set of markers, we selected 27,469 representative SNP sites in 10,105 target regions for the capture probe design. We performed the targeted enrichment sequencing with 100 F_1_ progeny segregating for starch viscosity phenotypes. Our results demonstrate that targeted enrichment by sequence capture is a reliable strategy to type SNPs in the coding regions. Even though the depth of coverage varied among target regions, it was consistent among individuals for given baits. We were able to identify 55,023 informative markers and generated the high-density SNP-based linkage map in cassava. This map contained 2,110 non-redundant SNP markers distributed over 19 linkage groups. We subsequently located the major QTL responsible for starch pasting time and temperature on LG 7, which coincided with location of the same QTL reported in Thanyasiriwat et al. [30]. We also discovered a novel QTL associated with starch pasting time on LG 10.

We have successfully expedited the process of QTL identification by using the multiplexed, targeted enrichment sequencing to genotype 100 segregating F_1_ progeny. Since a substantial number of SNPs were called from the targeted enrichment sequencing, the number of progeny genotyped can be scaled up in order to achieve the desired mapping resolution. Since coding regions are often more conserved than the rest of the genome, this set of capture probes are likely to be useful for other mapping populations, including those derived from interspecific crosses between *M.esculenta* and *M.glaziovii*.

## Supporting Information

S1 Fig **Distribution of sequence capture probes on the physical map.** Locations of the sequence capture probes were indicated on the physical map derived from the Cassava v5.0 Genome Assembly website (Available: http://www.phytozome.net/cassava.php. Accessed 2014 December 9.)
(PDF)

S1 Table **Statistics for targeted enrichment sequencing.** Quality scores measure the probability that the base is called incorrectly. Quality scores of 20 (Q20) represents a corresponding call accuracy of 99%.
(XLSX)

S2 Table **Detailed information of SNP markers on the linkage map.** The table displays the physical location of each SNP marker on the scaffolds (Cassava v5.0 Assembly Website. Available: http://www.phytozome.net/cassava.php. Accessed 2014 December 9.) and the position on the linkage groups. The LOD scores for pasting temperature (PT) and pasting time (PTi) were shown. The type of substitution was listed along with the codon and amino acid changes (if applicable).
(XLS)
