# Supplementary figures and images for: Large-Scale SNP Discovery through RNA Sequencing and SNP Genotyping by Targeted Enrichment Sequencing in Cassava (Manihot esculenta Crantz)
Source: PLoS One. 2014 Dec 31;9(12):e116028. doi: 10.1371/journal.pone.0116028 (PMC4281258; doi:10.1371/journal.pone.0116028)

# Probe locations

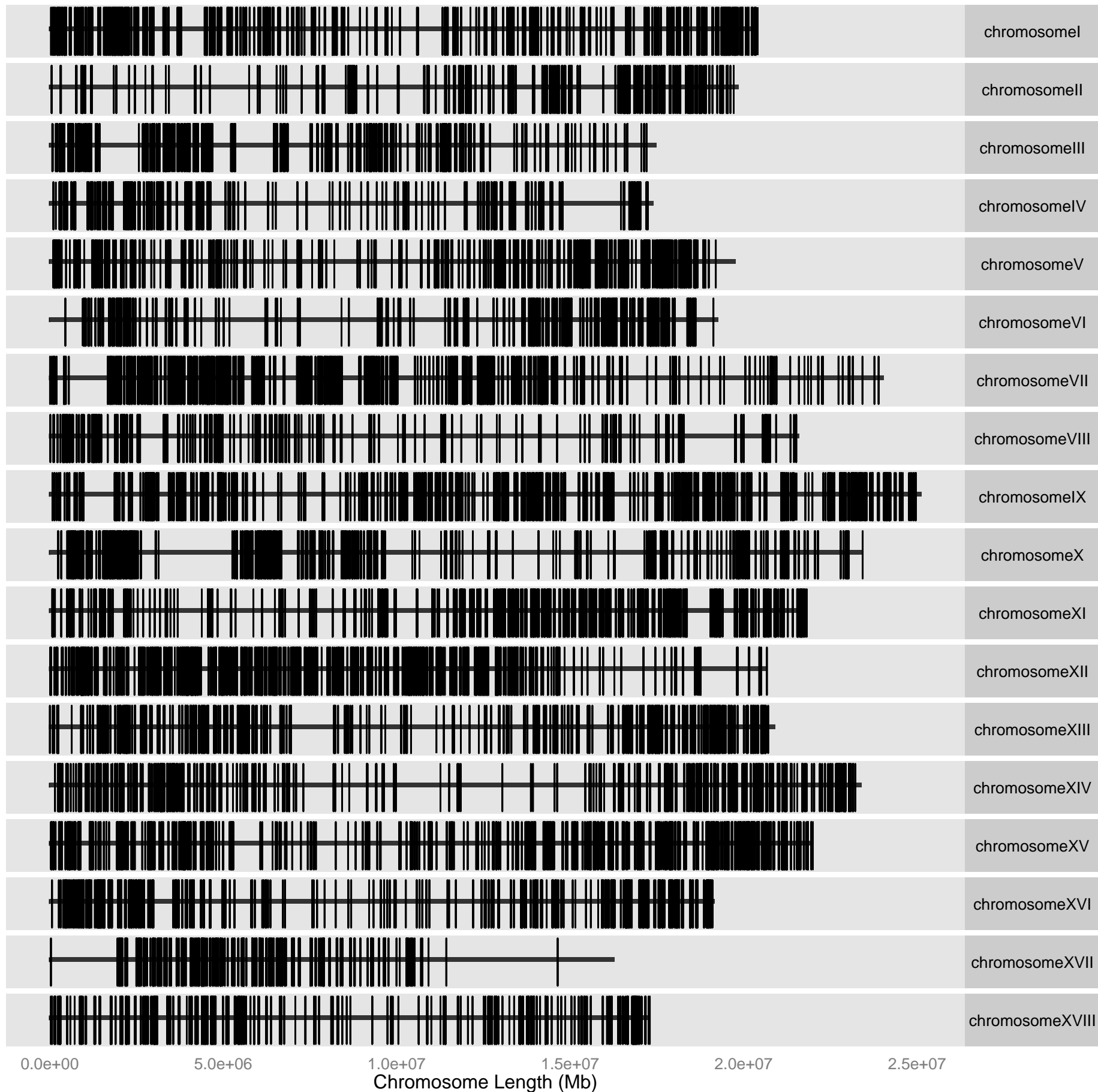

Supplement: S1 Fig — Distribution of sequence capture probes on the physical map. Locations of the sequence capture probes were indicated on the physical map derived from the Cassava v5.0 Genome Assembly website (Available: http://www.phytozome.net/cassava.php. Accessed 2014 December 9.) (PDF) [file pone.0116028.s001.pdf]
